# A Comparison of Dysautonomias Comorbid with Cyclic Vomiting Syndrome and with Migraine

**DOI:** 10.1155/2009/701019

**Published:** 2010-01-06

**Authors:** Gisela Chelimsky, Shruti Madan, Amer Alshekhlee, Elizabeth Heller, Kevin McNeeley, Thomas Chelimsky

**Affiliations:** ^1^Department of Pediatrics, University Hospitals of Cleveland, Cleveland, OH 44106, USA; ^2^Rainbow Babies Children's Hospital, University Hospitals Case Medical Center, Case Western Reserve University, Cleveland, OH 44106-6031, USA; ^3^Autonomic Laboratory, Neurological Institute, Cleveland, OH 44106-6031, USA

## Abstract

Cyclic vomiting syndrome (CVS) shares many features with migraine headache, including auras, photophobia, and antimigrainous treatment response being traditionally viewed as a migraine variant. *Aims*. To determine whether CVS is associated with the same disorders as migraine headache, and compare these associations to those in healthy control subjects. *Methods*. Cross-sectional study of patients utilizing the ODYSA instrument, evaluating the probability of 12 functional/autonomic diagnoses, CVS, migraine, orthostatic intolerance (OI), reflex syncope, interstitial cystitis, Raynaud's syndrome, complex regional pain syndrome (CRPS), irritable bowel syndrome, functional dyspepsia, functional abdominal pain, fibromyalgia, and chronic fatigue syndrome. Control subjects were age-matched gender-matched friends. Patients had to fulfill criteria for CVS or migraine, while control subjects could not. *Results*. 103 subjects were studied, 21 with CVS, 46 with migraine and 36 healthy controls. CVS and migraine did not differ in the relative frequencies of fibromyalgia, OI, syncope, and functional dyspepsia. However, CVS patients did demonstrate a significantly elevated frequency of CRPS. *Conclusions*. Although CVS and migraine clearly share many of the same comorbidities, they do differ in one important association, suggesting that they may not be identical in pathophysiology. Since OI is common in CVS, treatment strategies could also target this abnormality.

## 1. Introduction

Cyclic vomiting syndrome (CVS) is a condition characterized by stereotypical episodes of severe nausea and vomiting that last for several hours to several days, with return to baseline health between episodes [1]. CVS affects up to 2% of the population, and is described more commonly in children than in adults [2]. The episodes of vomiting vary in intensity and duration and are associated with pallor, increased salivation, listlessness, anorexia, nausea, retching, abdominal pain, headache, and photophobia. Many of these symptoms are thought to be generated by the autonomic nervous system and they are also present in patients with migraine [3]. CVS has no known etiology but a relationship to migraine is postulated based upon anecdotal similarities such as antecedent auras, associated headaches, and photophobia [4]. In addition, CVS is thought to respond reasonably to migraine therapy and one third of cases will evolve into migraines [5]. Furthermore, nausea or vomiting constitutes enough of a cornerstone of migraine presentation to be part of the criteria given by the International Headache Society [6]. Abdominal pain may also accompany migraine [7] but its clinical significance is unclear. Collectively, these symptoms may reflect involvement of the autonomic nervous system in migraine and CVS and hence suggest a common pathogenesis.

Migraine bears several well-described associations with other disorders such as fibromyalgia [8], irritable bowel syndrome [9], and syncope [10]. In this study, we show the spectrum of clinical similarities between subjects with migraine and CVS in comparison to control subjects.

## 2. Methods

This is a cross-sectional IRB approved review of the Ohio Dysautonomia (ODYSA) questionnaire. We enrolled subjects who fulfilled the criteria for CVS, migraine, and healthy control subjects.

Instrument. The ODYSA survey is a thorough clinical questionnaire designed to gather diagnostic information about 12 different autonomic disorders, which includes orthostatic intolerance, reflex syncope, CVS, interstitial cystitis, Raynaud's syndrome, complex regional pain syndrome (CRPS), irritable bowel syndrome, functional dyspepsia, functional abdominal pain, migraine headache, fibromyalgia, and chronic fatigue syndrome. The ODYSA questionnaire was developed to explore the extent to which these disorders are interrelated. The ODYSA instrument was developed over the last 6 years by a group of specialists including 2 urologists, 2 cardiologists, 2 neurologists, 1 adult gastroenterologist, 2 pediatric gastroenterologists, 1 rheumatologist, 2 geneticists, 1 epidemiologist, 1 psychologist, and 2 basic scientists with a particular interest in the dysautonomias within their field of expertise. We aimed to design and validate a clinical instrument able to noninvasively approximate the diagnosis of each of 12 dysautonomias. Where validated or published question-based diagnostic tools were available, we utilized these directly or modified them, as detailed below in Table 1, for each of the 12 disorders in the following table to create the ODYSA questionnaire. Although the resulting instrument is 19 pages long, it takes an average of only 16 minutes to complete, because we designed it to allow subjects to skip entire pages based on the lead question on the page, which ascertains whether any relevant symptoms are present.

## 3. Instrument

In the ODYSA questionnaire, a set of “probability” questions and a set of “severity” questions are embedded within the question set for each of the 12 disorders. The probability set is utilized to determine whether or not the responder has the disorder, while the severity set determines how disabling the disorder is to the subject. Only the probability set is assessed for evaluation of whether or not to include the subject in the group with the disorder. Probability scoring is forced to 0 or 1 (no intermediate values), while the severity score ranges from 0 to 10. These scores are programmed into a Filemaker Pro database to be generated automatically as each subject's data are entered. No observer input is required. Probability score determinations for comorbid disorders are listed below. The probability scores for CVS and migraine headache are listed under subject inclusions.

(1) *Orthostatic Intolerance*. The orthostatic intolerance probability criteria were validated against a tilt-table study demonstrating a positive and negative predictive values of 90.4% and 94.1% (unpublished data, manuscript in preparation).

(2) *Interstitial Cystitis*. We utilized 2 methods. (1) *O'Leary-Sant Method*. The validated and published O'Leary-Sant probability question set [21] addresses urinary urgency and frequency, nocturia, and pain or burning in the bladder. Scoring is performed by the program according to the original publication. (2) *NIDDK Method*. Additional questions were added following the NIDDK conference of 2006 to better assess the key diagnostic historical criteria of PBS such as pain that worsens with filling and that improves with voiding. These questions are face-valid as agreed in a teleconference with several experts in the field. Preliminary review of data suggested better reliability and validity of scoring method 2, which is the one currently in use.

(3) *Fibromyalgia* probability depends on the duration and location of pain that the subject experiences. The subject must experience pain for longer than 3 months, in question F2. Also, the subject has to assign a value to the pain ranging from 0 (no pain) to 5 (intolerable pain) in 21 specific areas of the body. These 21 points are divided into 4 quadrants: right upper, right lower, left upper, and left lower. Pain must be present in at least one site in each of the four quadrants of the body for the subject to have a probability of 1. Finally, the total score of the values assigned to each point must be greater than 4, meaning each quadrant has at least 1 area of tenderness. This set is face-valid. We also include a McGill Short Form [17], not utilized in this data set.

(4) *Reflex Syncope*. The probability of syncope relies on frequency and duration of loss of consciousness. Most of these questions were validated and published by Sheldon (2002, 2006) [15, 16]. For subjects to score a syncope probability of 1, they must be consciousness most of the time; unconsciousness must last less than 5 minutes, and it should take less than 1 hour until the subject returns to baseline awareness.

(5) *Irritable Bowel Syndrome* is based entirely on inclusion criteria and modular questions published in the Rome II book [11]. A subject must have experienced pain or discomfort in the center of the abdomen for at least 3 months, their discomfort or pain must improve after having a bowel movement, and the number and quality of bowel movements must change with the onset of pain or discomfort.

(6) *Functional Dyspepsia* is also based entirely on Rome II published criteria and modular questions [11]. Pain or discomfort must be centered in the upper abdomen with no evidence of an organic disease as likely cause, and no evidence that pain is relieved by defecation or associated with the onset of a change in stool frequency or form (which would qualify for IBS).

## 4. Subjects

Subjects were recruited from the autonomic laboratory, adult and pediatric clinics of different services including gastrointestinal, rheumatology, urology, autonomic, general neurology, and family medicine specialties at 3 tertiary care centers in Northeast Ohio. Each subject was asked to solicit their spouse or a friend to complete the survey as a control.

The inclusion criteria of CVS were adapted from The North American Society for Pediatric Gastroenterology, Hepatology and Nutrition clinical guidelines on the diagnosis and management of CVS [13]. These criteria included at least 3 episodes of intense vomiting and nausea in 6 months period or a total of 5 attacks in a lifetime. These episodes had to last from 1 hour to 10 days with a peak vomiting frequency of at least 4 emeses per hour and had to occur at least 1 week apart with a return to baseline health between episodes. Knowing that CVS may have a milder presentation, we also included a less severe patient group (“milder CVS”) who met all of the other criteria except that the peak vomiting frequency could be from 1–3 episodes per hour. The inclusion criteria for migraine were adapted from the International Headache Society [6] and these included 5 or more migraine attacks in a lifetime lasting between 4 to 72 hours (whether untreated or unsuccessfully treated). The attacks needed at least 2 of the following characteristics: unilateral location, pulsating quality, moderate or severe pain intensity, and aggravation by or causing avoidance of routine physical activity. During the attack, patients must have experienced at least 1 of the following: nausea and/or vomiting or photophobia and/or phonophobia and the headache could not be attributed to another disorder [6]. The control group included friends or spouses of the probands who did not meet ODYSA questionnaire criteria for CVS or migraine. We selected those control subjects in our database who were closest in age to the CVS subjects. The CVS and migraine subjects included any probands, family members or control subjects who met the ODYSA-based criteria for migraine or CVS.

Within the 3 groups we examined the following comorbidities: orthostatic intolerance, fibromyalgia, irritable bowel syndrome, complex regional pain syndrome, functional dyspepsia, syncope, and interstitial cystitis. Table 1 shows the source of the adapted inclusion criteria for these comorbid disorders. Chi-square and Fisher exact tests were used to compare the 3 categories and student's *t*-test was employed for continuous variables.

## 5. Results

A total of 103 subjects were included: 21 patients with CVS (3 children), of whom 7 were in the “milder CVS” group, 46 patients with migraine (3 children), and 36 healthy controls. In the CVS group, 15 subjects were probands (71%), 5 family members (24%) and 1 friend of a proband (5%). In the migraine group, 12 were probands (26%), 27 were family members (59%) and 7 friends of probands (15%). Female gender was predominant in each group [CVS (86%), migraine (85%), and healthy controls (64%)]. The mean age for those with CVS was similar to those with migraine, with a mean age of 41 ± 19.8 (range 6–68) for CVS and a mean age of 38.6 ± 14.1 (range 12–67) for the migraine group (*P* = .06). Healthy controls were about a decade younger with a mean age of 22.6 ± 13.9 (range 8–55). Table 2 summarizes the comorbidities present in each group. CVS and migraine subjects had similar comorbidities including fibromyalgia (38% in CVS and 22% in migraine; *P* = .12) and orthostatic intolerance (47% in CVS and 39% in migraine; *P* = .51). There was no difference in the rate of functional dyspepsia in the 2 groups (9.5% in CVS and 8.7% in migraine). Complex regional pain syndrome was more commonly reported in CVS than in migraine group, 23.8% versus 2.2%; *P* = .01.

**Figure 1:**
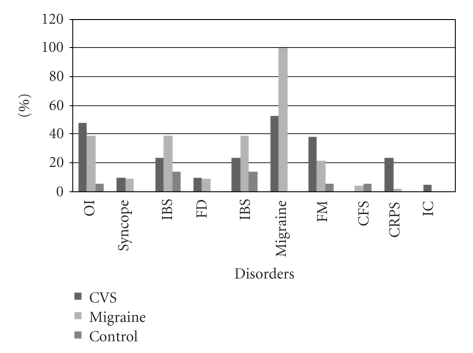
The range of autonomic co-morbidities in cyclic vomiting syndrome, migraine and healthy control groups.

More predominant autonomic comorbidities including orthostatic intolerance, fibromyalgia, and complex regional pain syndrome were found in the CVS group when compared to the control subjects. None of the controls had functional dyspepsia. The proportions of the coexisting autonomic comorbidities in the 3 groups are shown in Figure 1.

## 6. Discussion

Our study of CVS in a primarily adult population demonstrates three key points. First, adult CVS is clearly associated with other dysautonomias such as orthostatic intolerance, fibromyalgia, and complex regional pain. This association was limited to certain dysautonomias, and was not observed with others. For example, the rate of irritable bowel syndrome was not higher in CVS than in controls. These selective associations may ultimately provide clues to the pathogenesis of these disorders by demonstrating links between the disorders that share pathophysiologic elements and the disorders that do not. For instance, since CVS appears to be linked with orthostatic intolerance, the autonomic dysfunction known to play a role in orthostatic intolerance may also participate in the pathogenesis of CVS. Second, while significant similarities in comorbid disorders such as orthostatic intolerance, syncope, and functional dyspepsia clearly bridge CVS and migraine, other comorbidities are different. IBS, which was not associated with CVS, did demonstrate the expected association with migraine [9, 22]. IBS occurred in 18/46 (39%) subjects with migraines, compared to 5/36 control subjects (14%) (*P* = .004). The opposite finding was observed with CRPS which was more frequent in CVS than in migraineurs, as reported by others [23]. Taken together, this information supports a relationship between CVS and migraine but not as close as one might expect, at least in adults, if one disorder is truly just a variant of the other. Third, the significant association of both migraine and CVS with orthostatic intolerance suggests that these disorders could in fact respond to treatment directed at improving orthostatic tolerance as previously reported for other functional gastrointestinal disorders [24, 25]. This last hypothesis will require further evaluation.

The association of migraine with syncope has been previously highlighted in the CAMERA study, where syncope was more prominent in adults with migraine than in controls (46% versus 31% in controls) [10]. We have previously described the association of CVS with postural tachycardia syndrome and syncope [26] but this study was limited by referral bias. Patients had been enrolled from an autonomic laboratory, and their selection for referral for autonomic testing presumably reflected the predominance of their autonomic symptoms in the first place. In contrast, subjects in this study reflected a wide variety of referral sources, including nonspecialized clinics, the autonomic laboratory as well as family members of patients seen at the different institutions and friends. The high prevalence of orthostatic intolerance in the present study can therefore no longer be attributed to referral bias. The fact that this high prevalence was seen in both CVS and migraine is not surprising since it is also seen in other members of this group of disorders such as fibromyalgia [27], chronic fatigue [28], and irritable bowel [29]. Perhaps orthostatic intolerance constitutes the signature finding across all of these disorders, reflecting a common alteration in central autonomic network connectivity. 

This report is the first to report the association of CVS with fibromyalgia. This is not surprising as pain is a common denominator in children with functional gastrointestinal disorders and adults with chronic generalized pain. The co-occurrence of fibromyalgia and functional gastrointestinal disorders is recognized [29]. Therefore, the association of fibromyalgia with CVS, which is also a functional gastrointestinal disorder, fits with the current understanding of these disorders. It is striking that the two strongest associations of CVS, besides orthostatic intolerance, were both primarily chronic pain syndromes: fibromyalgia and CRPS. Although the difference between CVS and migraine in their associations with CRPS could reflect our small numbers (i.e., a type II error), this seems unlikely since the association occurred in the smaller CVS group where it has previously been reported, and did not occur in the larger migraine group. One would expect the opposite in a type II error, with the finding occurring in the larger group, and not in the smaller group where numbers are lacking. The association of CVS with chronic pain disorders might lead one to consider whether CVS itself might actually be better classified as a member of the group of chronic pain disorders, and whether the experience of nausea in this context may in fact be a variant of pain. Since the teleological purpose of nausea would presumably address some types of food poisoning, a form of tissue damage, the current International Association for the Study of Pain definition of pain would potentially include nausea: “An unpleasant sensory and emotional experience associated with actual or potential tissue damage, or described in terms of such damage.” Such a reconceptualization might suggest a more aggressive management approach, at least in adults, along the lines of a chronic pain syndrome. 

The current therapeutic recommendations for pediatric CVS in children older than 5 years of age include tricyclic antidepressants such as amitriptyline [13]. However, these agents may potentiate orthostatic intolerance due to their vasodilatory effect. Our results may provide additional guidance in the management strategy given such a high prevalence of orthostatic intolerance. Treatment aimed at orthostatic intolerance such as salt supplementation, fludrocortisones, or beta-adrenergic blockers should be clinically considered and further evaluated as therapy for CVS, both on their own strength and in combination with other agents such as the recommended tricyclic group. It should be kept in mind that these recommendations were aimed at a pediatric population, while the present study included predominantly adults. 

Our study has several limitations. First, we utilized a questionnaire to ascertain diagnoses. Although these question sets were based on specific criteria, the definitive diagnosis was not clinically determined through corroboration by physical examination or laboratory studies where appropriate. Second, autonomic studies were not performed on every patient in our study, and the sample of subjects tested is a partial subgroup of the total. Third, the control group was slightly younger than the migraine or CVS groups, though this is unlikely to affect the comorbidity findings, since CVS and migraine are disorders that tend to affect younger populations in general. However, we do not know whether adult CVS is the same disorder as childhood CVS, and the findings of this study may or may not apply to the pediatric population.

We conclude that while CVS and migraine share some autonomic comorbidities such as orthostatic intolerance, there are sufficient differences, at least in adults, to question the traditional teaching that CVS is a variant of migraine. In addition, the striking association of CVS with pain disorders such as CRPS and fibromyalgia suggests that it may share common pathogenic elements with these disorders.

## Figures and Tables

**Table 1:** Inclusion Criteria. This table shows the source of the adapted inclusion criteria for the comorbidities mentioned in ODYSA questionnaire.

Type of disease	Disease	Source
Bowel and bladder	Irritable bowel syndrome	Rome II criteria, 2nd. Ed., 2000 [11]
	Interstitial Cystitis	O'Leary-Sant Questions *Urology*, 1997, [12] Unmodified
	Cyclic vomiting syndrome	JPGN, 2008 [13]
	Functional Dyspepsia	Rome II criteria, 2nd. Ed., 2000 [11]
	Functional Abdominal Pain	Rome II criteria, 2nd. Ed., 2000 [11]

Cardiology	Orthostatic intolerance	Developed and partially validated by investigators, based on diagnostic criteria in Clinical Autonomic Disorders PA Low, 1997 [14]
	Reflex Syncope	Based on Question sets validated by Sheldon *JACC*, 2002; *Eur Heart J*, 2006 [15, 16]

Pain and fatigue	Migraine Headache	Face-valid questions developed by the investigators based on IHS Classification, *Cephalalgia*, 2004 [6]
	Fibromyalgia	Pain Score Localizer from JM Olson, 2001 McGill Short Form Questionnaire [17]
	Complex Regional Pain Syndrome/Reflex Sympathetic Dystrophy	Face-valid questions developed by the investigators based on IASP Criteria and Chelimsky, 1995 [18, 19]
	Chronic Fatigue Syndrome	Face-valid questions developed by the investigators based on CDC Criteria [20]
	Raynaud's syndrome	Face-valid questions developed by the investigators

**Table 2:** Autonomic co-morbidities in Cyclic Vomiting Syndrome (CVS), migraine, and healthy controls.

	CVS *n* = 21	Migraine *n* = 46	*P* value CVS versus migraine	Control *n* = 36	*P* value CVS versus controls
Females *n* (%)	18 (85.7)	39 (84.8)	.920	23 (63.9)	.08
Age mean ± SD	40.9 ± 19.8	38.6 ± 14.1	.058	22.64 ± 13.9	.077
Orthostatic intolerance *n* (%)	10 (47.6)	18 (39.1)	.51	2 (5.6)	.0002
Syncope *n* (%)	2 (9.5)	4 (8.7)	.91	0 (0)	.06
Functional dyspepsia	2 (9.5)	4 (8.7)	.88	0 (0)	.05
Irritable bowel syndrome *n* (%)	5 (23.8)	18 (39.1)	.22	5 (13.9)	.34
Migraine *n* (%)	11 (52.4)	46 (100)	—	0 (0)	—
Fibromyalgia *n* (%)	8 (38.1)	10 (21.7)	.12	2 (5.6)	.0015
Chronic fatigue syndrome *n* (%)	0 (0)	2 (4.3)	.34	2 (5.6)	—
Complex regional pain syndrome *n* (%)	5 (23.8)	1 (2.2)	.01	0 (0)	—
Interstitial cystitis	1 (4.8)	0 (0)	Not run	0 (0)	Not run

## Abbreviations

CVS: Cyclic vomiting syndrome;
OI: Orthostatic intolerance;
IBS: Irritable bowel syndrome;
FD: Function dyspepsia;
FM: Fibromyalgia;
CFS: Chronic fatigue syndrome;
CRPS: Complex regional pain syndrome;
IC: Interstitial cystitis.
